# Longitudinal assessment of DNA recovery from post-mortem whole blood stored in EDTA, sodium fluoride/potassium oxalate and additive-free tubes

**DOI:** 10.1007/s00414-024-03384-z

**Published:** 2024-12-10

**Authors:** Jana Grobbelaar, Loyiso Abongile Marvin Vuko, Bronwen Davies, Brendon Pearce, Fungisai Lorraine Musiyandaka, Laura Jane Heathfield

**Affiliations:** 1 Division of Forensic Medicine and Toxicology, Department of Pathology, Faculty of Health Sciences, University of Cape Town, Anzio Road, Cape Town, South Africa; 2 Genetics Department, Faculty of Agriscience, Stellenbosch University, Van Der Bijl Street, Stellenbosch, South Africa

**Keywords:** Molecular autopsy, Drug death investigation, Whole blood storage, Salting-out DNA extraction, Forensic pathology

## Abstract

Adverse drug reactions and fatalities can result from therapeutic drug use due to genetic deficiencies in drug-metabolizing enzymes. In cases where ancillary testing may not reveal a clear cause of death, molecular autopsies can be valuable. However, forensic mortuaries do not retain DNA samples in all cases, which hinders subsequent genetic testing if it is later deemed necessary. This study aimed to evaluate whether post-mortem whole blood samples collected for toxicological analysis, could provide viable DNA for genetic testing after varying storage periods. Thirty deceased individuals were recruited with informed consent. Blood collected at autopsy from each individual was stored in two sodium fluoride/potassium oxalate (gray-top) tubes, two additive-free (red-top) tubes and one ethylenediaminetetraacetic acid (EDTA; purple-top) tube– the latter recommended for DNA analysis. Blood from one gray-top and one red-top tube were sampled for toxicological analysis prior to DNA analysis, while the remaining samples (acting as controls) underwent DNA analysis immediately. DNA analysis involved DNA extraction and DNA concentration and degradation assessment. Blood samples were stored at 4 °C and DNA extraction and analysis was repeated one year and then five years later. Toxicological sampling did not significantly influence DNA results. DNA concentration and quality significantly decreased over time for all sample types, with DNA from red-top tubes showing the greatest decline. The study showed that DNA testing for drug-metabolizing enzymes was feasible on whole blood that had been stored for five years. This finding supports the potential for retrospective genetic testing in cases of adverse drug reactions and fatalities.

## Introduction

The administration and misuse of scheduled drugs is a frequent and expanding concern worldwide [1]. Drug misuse is particularly concerning, as it involves the intake of drugs in ways other than originally intended, including prescription nonadherence, incorrect dosing, using an improper route of administration, or using drugs prescribed to another person [2]. Consequently, drug misuse may lead to reduced medication effectiveness, increased healthcare expenses, additional comorbidities, hospitalizations, and even death [2]. The most recent available data (2019) suggests that approximately 500 000 unnatural deaths globally were attributed to the abuse of drugs, which represents an overall increase of 17.5% compared to 2009 [1].

While most drug-induced fatalities result from accidental or intentional overdoses [3], there are instances where idiosyncratic adverse drug reactions can lead to death, even with non-lethal drug doses [4]. Genetic polymorphisms, specifically those within hepatic enzyme genes like the cytochrome P450 (CYP) enzyme system (CYP1, 2, and 3 families), have been shown to play a role in these deaths [5], and are recognized in forensic toxicology applications [6].

Of particular interest is the highly polymorphic enzyme CYP2D6, which plays a role in metabolizing approximately one quarter of commonly used drugs [7]. The expression of CYP enzymes is influenced by age, sex, ethnicity, and disease status [4]. Unlike other CYP enzymes, CYP2D6 is unlikely to be induced by external factors like xenobiotics, hormones, or environmental influences [5]. This makes it a significant contributor to individual variations in drug metabolism and toxicity, driven by population-specific genetic differences that determine metabolic phenotypes [7]. These variations contribute to four distinct metabolic phenotypes [8]: poor metabolizers carry non-functional alleles or gene deletions; intermediate metabolizers have reduced function alleles; extensive metabolizers possess fully functional alleles; and ultrarapid metabolizers carry gene duplications or activity-enhancing mutations.

Drug-induced fatalities often present non-distinct pathologies at the time of autopsy, leaving the cause of death undetermined [9]. Ancillary investigations, such as histology and toxicology, may be performed when the gross autopsy fails to provide definitive answers [6]. Whole blood collected from peripheral sites is the preferred specimen for quantitative analysis and subsequent interpretation, and its proper storage is crucial for preserving analytes [9]. Sodium fluoride/potassium oxalate (gray-top) tubes (0.5-2% w/v) are commonly used to maintain analyte stability [10], but in cases where an additive might interfere with analysis, additive-free (red-top) tubes are employed [9]. Forensic toxicologists then employ various analytical tests, including chromatography and mass spectrometry, to detect, identify and quantify drugs, metabolites, and chemicals. The determination of drug and/or metabolite concentrations in blood and other specimens is typically crucial in establishing its role in the cause of death within the case context [11].

The interpretation of toxicological results is not always straightforward, however. Factors like overlapping drug concentration ranges (therapeutic versus toxic) in blood and high or low parent to metabolite (P/M) drug ratios can complicate understanding of results [12]. Most notably, a high P/M ratio in blood may indicate acute overdose or recent drug consumption, but it can also result from drug interactions or genetic variations in drug metabolism [13]. Determining the manner of death in drug-related cases, especially those involving affected drug metabolism due to genetics, can be challenging [14]. Toxicology results may suggest that drug use caused or contributed to death, but without relevant medical and case history, the intent may remain unclear [14]. Complete autopsies and ancillary investigations might yield ambiguous results, making it difficult to definitively determine the cause and manner of death [6].

In these cases, a molecular autopsy may be incorporated to identify a potential underlying genetic cause of death, potentially aiding in reducing the number of unresolved cases [6]. Various case reports have highlighted that genetic testing may be useful in selected drug-related death cases [14–16], indicating that molecular autopsies targeting variations in the genes encoding drug-metabolizing enzymes such as CYP2D6 may be a valuable interpretive aid in both clinical and medico-legal settings. The gold standard method for sample collection for postmortem genetic screening involves drawing blood into 1–2 mg/mL ethylenediaminetetraacetic acid (EDTA) tubes [17]. Molecular autopsies are, however, not routinely conducted during medico-legal investigations, as the need for DNA testing often becomes apparent only after the completion of the autopsy and toxicological analysis [17], and many forensic laboratories do not have standard operating procedures relating to genetic testing for drug death investigations [18, 19]. The blood specimens taken for toxicological testing may then be the only samples available for further testing, which were not necessarily collected or handled with DNA preservation in mind. South African forensic chemistry laboratories have previously reported up to a 10-year backlog in sample processing [20], which raises the question of whether samples intended for toxicology testing and stored long-term would be suitable for genetic testing, if it is later deemed necessary.

To the authors’ knowledge, no studies have investigated whether the handling of blood in a toxicology laboratory environment compromises DNA quality and quantity, or whether usable DNA sequencing data can be obtained from whole blood that was stored in gray- and red-top tubes for extended periods. To address this knowledge gap, this longitudinal study aimed to investigate whether blood specimens that were collected in gray- and red-top tubes and handled in a DNA-uncontrolled laboratory can still yield DNA of sufficient quality and quantity to perform genetic testing after a prolonged storage period.

## Methods and materials

### Study population

A cohort of 30 post-mortem cases admitted for an autopsy to Salt River Mortuary in Cape Town, South Africa between 1 May 2017 and 30 August 2017 were recruited in 2017. Written informed consent for the collection of blood samples from the deceased individuals was obtained, which adhered to published ethical considerations [21]. The final cohort consisted of 22 males between the age of 22 years and 66 years, and 8 females between the age of 21 years and 57 years. Cases were included in the study if drug toxicity was a suspected primary or contributory cause of death, or if the case was a sudden or unexpected death in adults (SUDA), where the decedent had a reported history of drug use. Cases were excluded from the study if the decedent was below the age of 18 or if the body was severely burnt or decomposed.

### Sample collection

Femoral blood (20 mL) was collected from each of the 30 cases at the time of autopsy. Samples were aliquoted in 4 mL volumes into one purple-top vial containing EDTA (BD Vacutainer, New Jersey, USA), two gray-top tubes with sodium fluoride and potassium oxalate (SG Vac, Johannesburg, SA), and two red-top tubes (no additive) (SG Vac, Johannesburg, SA) for each of the cases. Each case and their samples were anonymized through unique case identification numbers to ensure confidentiality.

### DNA extraction (silica column)

Within 72 h after sample collection, DNA was extracted from the purple-top vial, one gray-top vial and one red-top vial for each case (*n* = 90). These samples did not undergo toxicological analysis and were used as controls (termed herein as P-No-Tox, G-No-Tox, and R-No-Tox, respectively). The remaining gray-top and red-top tubes for each case (termed herein as G-Tox and R-Tox, respectively) were prepared for analysis in the UCT Forensic Toxicology Unit laboratory and submitted for toxicological screening at the UCT Division of Pharmacology prior to DNA analysis (*n* = 60). DNA was extracted from the 150 stored blood samples using the QIAamp^®^ DNA Investigator kit (Qiagen, Hilden, Germany) following the manufacturer’s protocol, with slight adjustments as follows: A total of 100 µL of each blood sample was utilized, and the purified DNA was eluted into 50 µL of Qiagen^®^ ATE Elution Buffer and centrifuged at full speed (20 238 rcf) for an extended period of 90 s. This elution step was repeated, resulting in two eluates of extracted DNA for each sample. Following DNA extraction, blood samples were stored at 4 °C until the one-year and five-year time points, after which the DNA extraction process was repeated using the same protocol. At the five-year time point, 13 of the blood samples had dried, and were reconstituted in 2 mL 1X phosphate-buffered saline (Thermo Fisher Scientific, Massachusetts, USA) according to the method described by Tagliaferro et al. [22]. Molecular biology grade water (Thermo Fisher Scientific, Massachusetts, USA) stored in identical purple, gray, and red-top tubes underwent the same DNA extraction process to serve as negative controls.

### DNA extraction (salting-out)

After five years, the procedure described above was no longer sufficient to recover usable DNA for Sanger sequencing. Therefore, the salting-out DNA extraction procedure developed by Miller et al. [23] was assessed, optimized, and applied to the 150 samples. A total of 2 mL of whole blood from each sample was digested with 12 mL of red blood cell lysis buffer (8.28 g/L > 99% ammonium chloride (Kimix, Durban, South Africa), 0.79 g/L > 99% ammonium bicarbonate (Sigma-Aldrich, Missouri, USA), 0.5 M EDTA at pH 7.4 (Thermo Fisher Scientific, Massachusetts, USA)) and centrifuged at 376 rcf for 20 min, after which the supernatant was discarded. The red blood cell digestion step was repeated a total of four times. The resulting cell pellet was then resuspended in 1 mL white blood cell lysis buffer (1 M Tris-HCl at pH 7.5 (Lonza, Basel, Switzerland), 0.5 M EDTA at pH 7.4 (Thermo Fisher Scientific, Massachusetts, USA), 3 M sodium chloride (Merck, New Jersey, USA)). The lysate was incubated with 40 µL of 20% SDS (Merck, New Jersey, USA) and 40 µL of 20 mg/mL Proteinase K (Qiagen, Hilden, Germany) for 24 h at 56 °C with shaking at 211 rcf.

Following overnight incubation, an equivalent amount of 6 M sodium chloride (Merck, New Jersey, USA) was added to the lysate and the samples were incubated at -20 °C for 30 min. The samples were then centrifuged at maximum speed (20 238 rcf) for 15 min, after which the resulting pellet was discarded, and the supernatant was transferred to a clean tube containing an equivalent amount of > 99.9% HPLC grade isopropanol (Sigma-Aldrich, Missouri, USA). For samples where the supernatant was not clear following centrifugation, the supernatant was transferred to a clean tube and centrifuged for an additional 10 min at 6010 rcf before being added to the absolute isopropanol. The samples were then incubated at -80 °C for 30 min and centrifuged at maximum speed for 15 min. The resulting supernatant was decanted, and the DNA pellet was washed twice with 70% ethanol (Merck, New Jersey, USA), with a 5 min centrifugation step at 15 871 rcf separating the washes. Finally, the DNA pellet was dried at 37 °C and resuspended in 50 µL of 1X Tris-EDTA buffer (Merck, New Jersey, USA).

An additional purification step using sodium acetate was tested on a subset of DNA extracts, but this did not improve results and was not tested further.

**Fig. 1:**
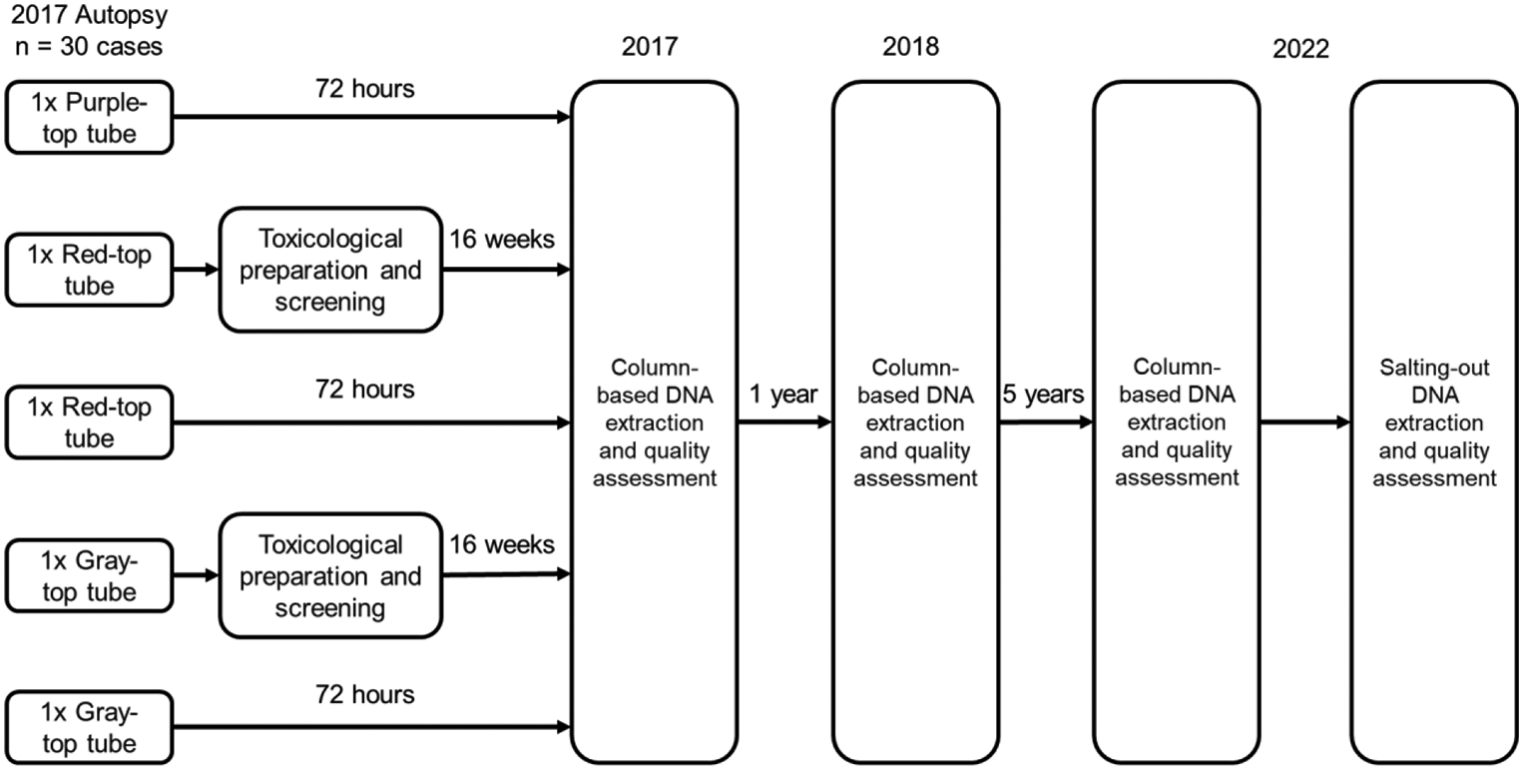
Illustration of the procedures followed during sample collection and subsequent laboratory processing between 2017 and 2022

A summary of the sample collection and laboratory procedures followed throughout the longitudinal study is depicted in Fig. 1.

### Assessment of DNA quantity and quality

#### Real-time PCR (qPCR)

DNA quantity and quality were further assessed by real-time PCR using the Quantifiler^®^ Trio DNA Quantification kit (Thermo Fisher Scientific, Massachusetts, USA) according to the manufacturer’s protocol. This was preceded by NanoDrop™ spectrophotometry to determine whether samples required dilution to a concentration below 50 ng/µL to not exceed the upper limit of the standard curve. Amplification was performed on the 7500 Real-Time PCR system (Applied Biosystems, California, USA). The concentration of the large autosomal target was used as the final DNA concentration. An identical reaction mixture lacking template DNA functioned as a negative control. As part of the kit, a synthetic DNA template amplified alongside each sample as an internal positive control. Real-time PCR was also used to determine the ‘degradation index’ (DI). The DI values were interpreted according to the method described by Vernarecci et al. [24].

#### Forensic DNA profiling

In 2017, DNA profiling was performed to assess if samples that had undergone sampling for toxicological analysis in a DNA-uncontrolled laboratory had been contaminated with extraneous DNA. The PowerPlex^®^ ESI 16 System (Promega, Wisconsin, USA) was used to prepare quarter-volume multiplex PCR reactions with 0.5 ng/µl template DNA according to the manufacturer’s recommendations. Identical reaction mixtures lacking template DNA functioned as negative controls. The multiplex system amplified the following loci: Amelogenin, D3S1358, D19S433, D2S1338, D22S1045, D16S539, D18S51, D1S1656, D10S1248, D2S441, TH01, vWA, D21S11, D12S391, D8S1179, and FGA.

Following PCR, each sample was added into an Applied Biosystems MicroAmp^®^ optical 96-well plate containing WEN Internal Lane Standard 500 and Hi-Di™ formamide (Promega, Wisconsin, USA) according to the manufacturer’s instructions. Samples were run on an Applied Biosystems Genetic Analyser 3130 *xl* at 60 °C and results were analyzed using Applied Biosystems GeneMapper version 4.1 software (Applied Biosystems, California, USA).

### Genetic analysis

Sanger sequencing was employed to assess whether usable sequencing data could be obtained following long-term storage in the various vial types, and not to determine the presence of variants or the phenotype of the individual. The nine coding regions of *CYP2D6* (target A-I) were amplified using 2X GoTaq Green Master mix (Promega, Wisconsin, USA) and 10 µM of each primer (Table 1) in a total volume of 25 µl. Identical PCR reaction mixtures lacking template DNA to a final volume of 25 µL, functioned as negative control specimens. Agarose gel electrophoresis was utilized to determine whether amplification was successful. Prior to sequencing of the amplified products, a post-PCR clean-up step was performed with the Nucleofast^®^ 96 PCR Clean-up kit (Macherey-Nagel, Düren, Germany) as outlined in the manufacturer’s instructions. Bi-directional sequencing was performed at the Central Analytical Facility of the University of Stellenbosch, South Africa using the BigDye^®^ Terminator v3.0 cycle kit (Applied Biosystems, California, USA) according to their in-house protocol. The 15 resulting sequences were then compared to the *CYP2D6* reference sequence available on the Ensembl genome browser Release 108 (http://www.ensembl.org; accessed 23 November 2022) using the BioEdit Sequence Alignment Editor version 7.2.5 [25] and ClustalW with 1000 bootstrap. Sequences were deemed unusable if nucleotide calling was incomplete or alignment to the reference sequence was not possible. Poor quality sequences had a high signal-to-noise ratio, and numerous chemical artefacts or errors in base pair calling, resulting in partial alignment to the reference sequence. Good quality sequences had a low signal-to-noise ratio, complete base pair calling and full alignment to the reference sequence.

**Table 1:** Primer sets and their corresponding *CYP2D6* target regions

Target	CYP2D6 exon/s	Primer sequence (5’-3’)	Primer direction
A^1^	Part of 1	GCCATCATCAGCTCCCTT	Forward
		CCCAAACCTGCTTCCCCTT	Reverse
B^1^	Part of 1	CCCTACCAGAAGCAAACA	Forward
		CCTATTTGAACCTTGGACGA	Reverse
C^1^	Part of 1	CTTCCACCTGCTCACTCC	Forward
		TCTGTCTCTGTCCCCACC	Reverse
D^2^	2 and 3	GTGGATGGTGGGGCTAAT	Forward
		ACTCCTCGGTCTCTCGCT	Reverse
E	4	CCCGTTCTGTCTGGTGTAG	Forward
		AGCCTCCCCTCATTCCTC	Reverse
F^3^	5 and 6	GTTCTGTCCCGAGTATGC	Forward
		CCTGACACTCCTTCTTGC	Reverse
G	7	CATAGGAGGCAAGAAGGAG	Forward
		TGGTGGCATTGAGGACTA	Reverse
H	8	ATCCTAGAGTCCAGTCCC	Forward
		ACTACCACATTGCTTTATTGTAC	Reverse
I	9	TATCACCCAGGAGCCAGG	Forward
		CCCACATGCCAGGACAAT	Reverse

^1^ Targets A, B and C are not full-length exons, but regions within exon 1 of the *CYP2D6* gene

^2^ Target D incorporates the full-length exons 2 and 3 of the *CYP2D6* gene

^3^ Target F incorporates the full-length exons 5 and 6 of the *CYP2D6* gene

Based on the results of the toxicological analysis, Case 22 was selected for genetic analysis due to the detection of drugs that are metabolized by the CYP2D6 enzyme. This case involves the death of a 33-year-old male from a suspected accidental overdose. The decedent allegedly had a strong history of substance abuse and was discharged from a rehabilitation center four months prior to his death. An overdose was suspected as the decedent was allegedly found with a needle in his hand and other drug paraphernalia on scene. The postmortem examination indicated a puncture wound on the right arm, blood and vomitus in and around the mouth, and features of gastric aspiration and hepatosplenomegaly. Toxicological analysis detected the presence of methaqualone in the blood and amphetamine, methamphetamine, morphine-3-ß-D-glucuronide, 6-O-monoacetylmorphine, methaqualone, diphenhydramine, cocaine and benzoylecgonine in the urine.

DNA extracted samples from blood that had undergone toxicological sampling and screening for Case 22 were used (22G-Tox and 22R-Tox). DNA from the 22G-Tox and 22R-Tox samples extracted using the modified salting-out method was also analyzed at the five-year time point.

### Data analysis

Statistical analysis was performed using the IBM SPSS Statistics Software version 28.0.1 (SPSS Inc., Illinois, USA) at a level of significance (α) of 0.05. The distributions of the data were assessed using the Shapiro-Wilk test. The data were analyzed using the Related-Samples Friedman’s Two-Way Analysis of Variance by Rank test. Effect sizes were determined using the Kendall’s W test. The main effects of vial type and time since sample collection were assessed individually to determine whether a significant difference exists in terms of DNA concentration, purity ratios and degradation index. There was no significant difference between Tox and No-Tox samples, therefore these results were combined when analyzing the effect of time since sample collection. Familywise errors were accounted for using the Bonferroni correction on multiple comparisons. To assess whether the modified salting-out DNA extraction method significantly increased DNA yield and sample purity overall and within the three tube types, a Related-Samples Wilcoxon Signed Rank test was utilized. Effect sizes were calculated by dividing the standardized test statistic Z with the square root of the number of comparisons.

## Results

### DNA concentration (column-based method)

**Fig. 2:**
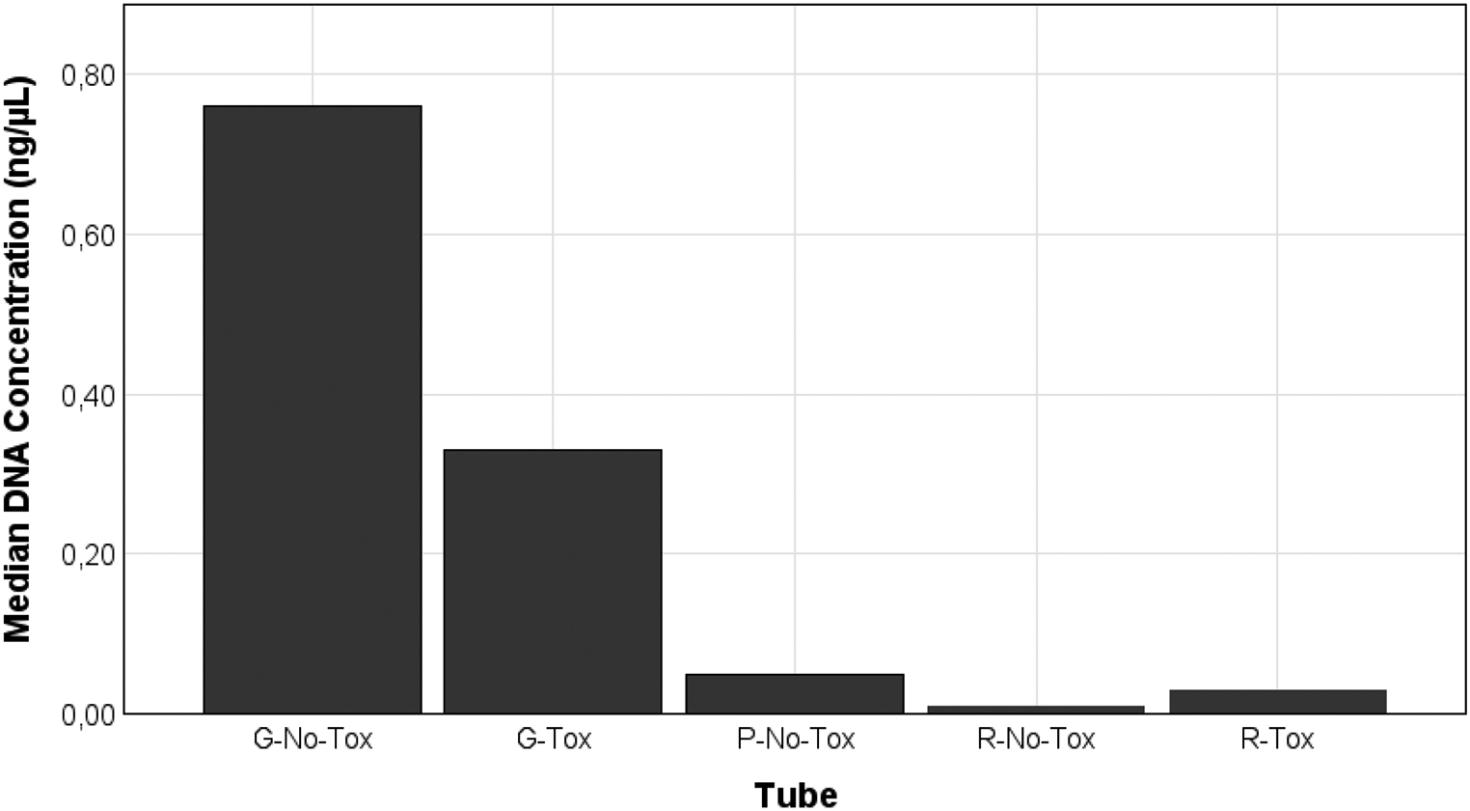
Comparison of the median DNA concentrations (ng/µL) obtained after a five-year storage period between gray (G), purple (P), and red-top (R) tubes that had and had not undergone toxicological processing prior to DNA extraction using a column-based method (Tox and No-Tox, respectively). Concentrations were determined using real-time PCR

After five years, a significant difference in median DNA concentrations were found between the groups (x^2^ = 55.343; W = 0.494; *p* < 0.001). Gray-top tubes showed the highest median DNA concentrations, with 0.76 ± 11.93 ng/µL and 0.33 ± 8.93 ng/µL for the G-No-Tox and G-Tox groups, respectively. Red-top tubes showed the lowest median DNA concentrations, with 0.03 ± 0.07 ng/µL for the R-Tox group and 0.01 ± 0.21 ng/µL for the R-No-Tox group. Toxicological preparations did not significantly influence DNA concentrations obtained between these groups for both the gray-top and red-top tubes (*p* = 1.00). The P-No-Tox group showed a median concentration of 0.05 ± 4.62 ng/µL, which was significantly higher than the R-No-Tox group (*p* = 0.018), but not significantly different from the G-Tox (*p* = 0.346) and R-Tox (*p* = 0.759) groups. The G-Tox median concentration was significantly higher compared to the R-Tox (*p* = 0.001) and R-No-Tox (*p* < 0.001) groups. The G-No-Tox median concentration was significantly higher than the R-Tox (*p* < 0.001), R-No-Tox (*p* < 0.001) and P-No-Tox (*p* = 0.013) groups. Median concentrations for each group are depicted in Fig. 2.

**Fig. 3:**
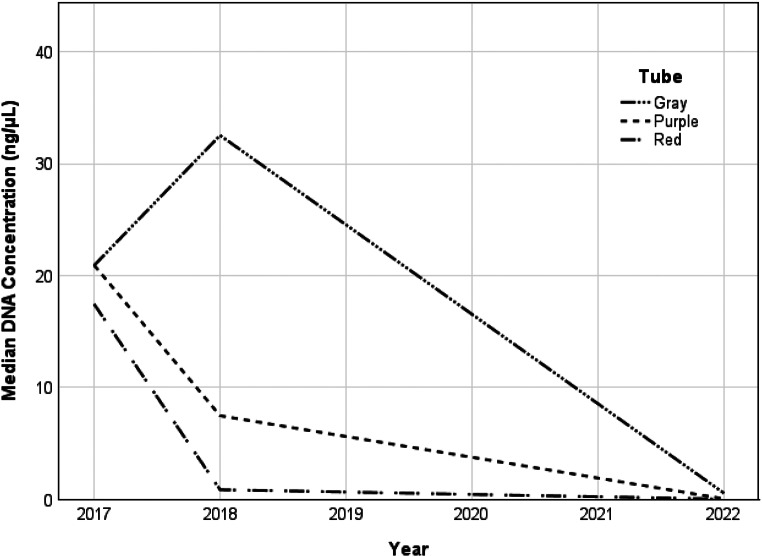
Comparison of the median DNA concentrations (ng/µL) obtained between gray (G), purple (P), and red-top (R) tubes immediately (2017), one year (2018) and five years (2022) after sample collection. Concentrations were determined using real-time PCR

The changes in median DNA concentration over time for each tube type were assessed (Fig. 3). Gray-top tubes showed significant differences over time (x^2^ = 73.379; W = 0.633; *p* < 0.001) with a collective median concentration of 20.9 ± 14.31 ng/µL in 2017, 33.2 ± 41.73 ng/µL in 2018, and 0.58 ± 10.3 ng/µL in 2022. The median concentration increased between 2017 and 2018 but was not significant (*p* = 0.19). A significant decrease in median concentration was observed between 2017 and 2022 (*p* < 0.001) and 2018–2022 (*p* < 0.001). Red-top tubes showed significant differences over time (x^2^ = 108.138; W = 0.932; *p* < 0.001) with a collective median concentration of 17.45 ± 36.36 ng/µL in 2017, 0.94 ± 5.54 ng/µL in 2018 and 0.02 ± 0.15 ng/µL in 2022. This decrease was significant between 2017 and 2018 (*p* < 0.001), 2017–2022 (*p* < 0.001) and 2018–2022 (*p* < 0.001). Purple-top tubes also showed significant differences over time (x^2^ = 42.276; W = 0.729; *p* < 0.001) with a collective median concentration of 22.32 ± 15.35 ng/µL in 2017, 7.47 ± 29.77 ng/µL in 2018 and 0.05 ± 4.62 ng/µL in 2022. A significant decrease in concentration was seen between 2017 and 2022 (*p* < 0.001) and 2018–2022 (*p* < 0.001). The decrease between 2017 and 2018 was not significant (*p* = 0.567).

### Degradation index (DI)

**Fig. 4:**
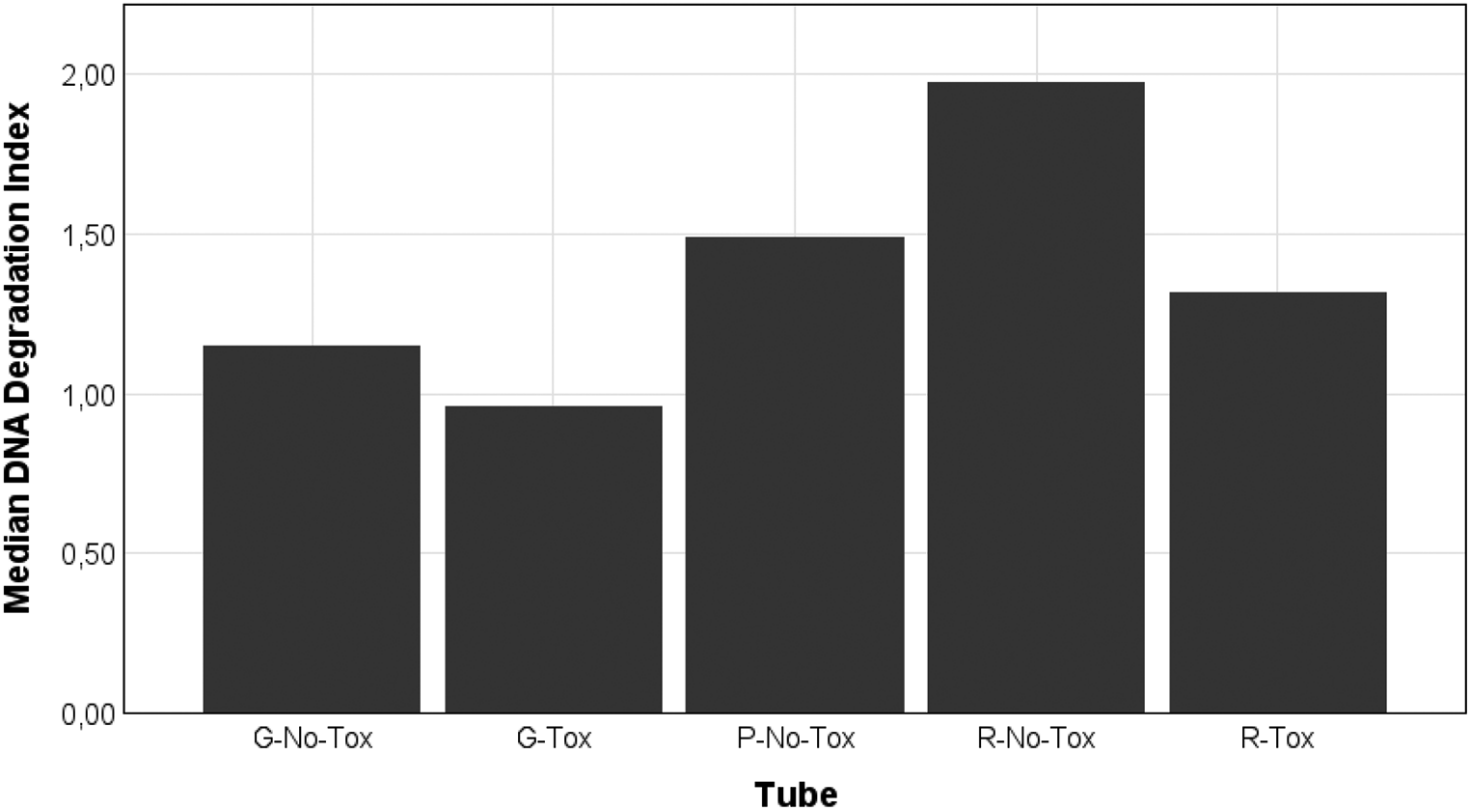
Comparison of the median degradation indices observed after a five-year storage period between gray (G), purple (P), and red-top (R) tubes that have and have not undergone preparations for toxicological processing prior to DNA extraction using a column-based method (Tox and No-Tox, respectively). Degradation indices were determined using real-time PCR

No significant differences were found between the groups after 5 years (x^2^ = 6.311; W = 0.058; *p* = 0.177). The R-No-Tox group showed a higher DI (median = 1.98, SD = 1.78) compared to the following groups: P-No-Tox (median = 1.49, SD = 2.12, *p* = 1.00), R-Tox (median = 1.32, SD = 0.74, *p* = 1.00), G-No-Tox (median = 1.15, SD = 2.27, *p* = 0.201), and G-Tox (median = 0.96, SD = 3.85, *p* = 0.852) following five years of storage. The P-No-Tox group median DI was higher compared to the G-No-Tox (*p* = 1.00), G-Tox (*p* = 1.00), and R-Tox (*p* = 1.00) groups. The R-Tox group median DI was higher than that of the G-No-Tox and G-Tox groups (*p* = 1.00, respectively). The G-No-Tox group median DI was higher than that of the G-Tox group (*p* = 1.00). None of these comparisons were significant. Median DI values for each group are depicted in Fig. 4.

Of the gray-top tube samples, 37 were undegraded, 15 were mildly degraded, 4 were degraded and 3 were severely degraded. Of the red-top tube samples, 28 were undegraded, 24 were mildly degraded and 5 were degraded. Of the purple-top tube samples, 15 were undegraded, 10 were slightly degraded, 4 were degraded and 1 was severely degraded. These levels of degradation have been described previously by Vernarecci et al. [24].

**Fig. 5:**
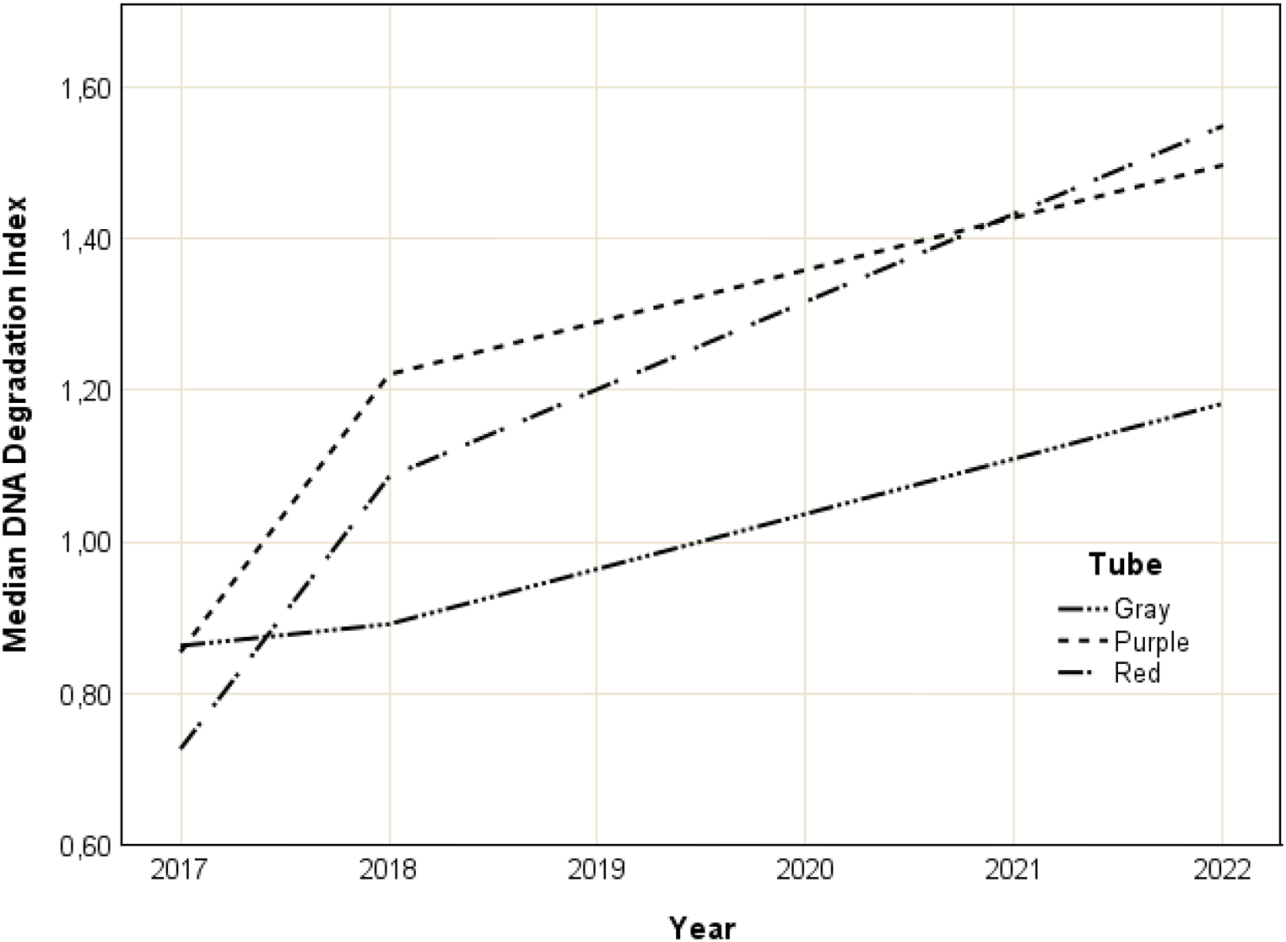
Comparison of the median degradation indices observed between gray (G), purple (P), and red-top (R) tubes immediately (2017), one year (2018) and five years (2022) after sample collection. Degradation indices were determined using real-time PCR

The changes in median DI over time for each tube type were assessed (Fig. 5). Over time, gray-top tubes showed significant differences in DI (x^2^ = 8.172; W = 0.07; *p* = 0.017). In 2017, gray-top tubes showed a median DI of 0.86 ± 0.13, compared to 0.89 ± 1.79 in 2018 and 1.19 ± 6.00 in 2022. The median DI of gray-top tubes significantly increased between 2017 and 2022 (*p* = 0.016). The increases between 2017 and 2018 (*p* = 1.00) and 2018–2022 (*p* = 0.154) were insignificant. The median Di of red-top tubes differed significantly over time (x^2^ = 50.667; W = 0.444; *p* < 0.001). Red-top tubes showed a median DI of 0.72 ± 0.39 in 2017, 1.09 ± 1.36 in 2018 and 1.55 ± 1.39 in 2022. This increase was significant between 2017 and 2018 (*p* < 0.001) and 2017–2022 (*p* < 0.001) but insignificant between 2018 and 2022 (*p* = 0.118). The median DI of purple-top tubes showed significant differences over time (x^2^ = 16.828; W = 0.29; *p* < 0.001) with an increase from 0.87 ± 0.30 in 2017 to 1.22 ± 1.69 in 2018 and 1.49 ± 3.33 in 2022, which was significant between 2017 and 2018 (*p* = 0.002) and 2017–2022 (*p* = 0.001). The increase between 2018 and 2022 was insignificant (*p* = 1.00).

### Forensic DNA profiling

No DNA profile mixture was observed in any of the electropherograms examined for this analysis, despite being handled in a DNA-uncontrolled laboratory. Furthermore, complete DNA profiles could be obtained using DNA extracted from both red- and gray-top tube samples. Full DNA profiles are not shown to protect the identity of the participants.

### Comparison of extraction methods

**Fig. 6:**
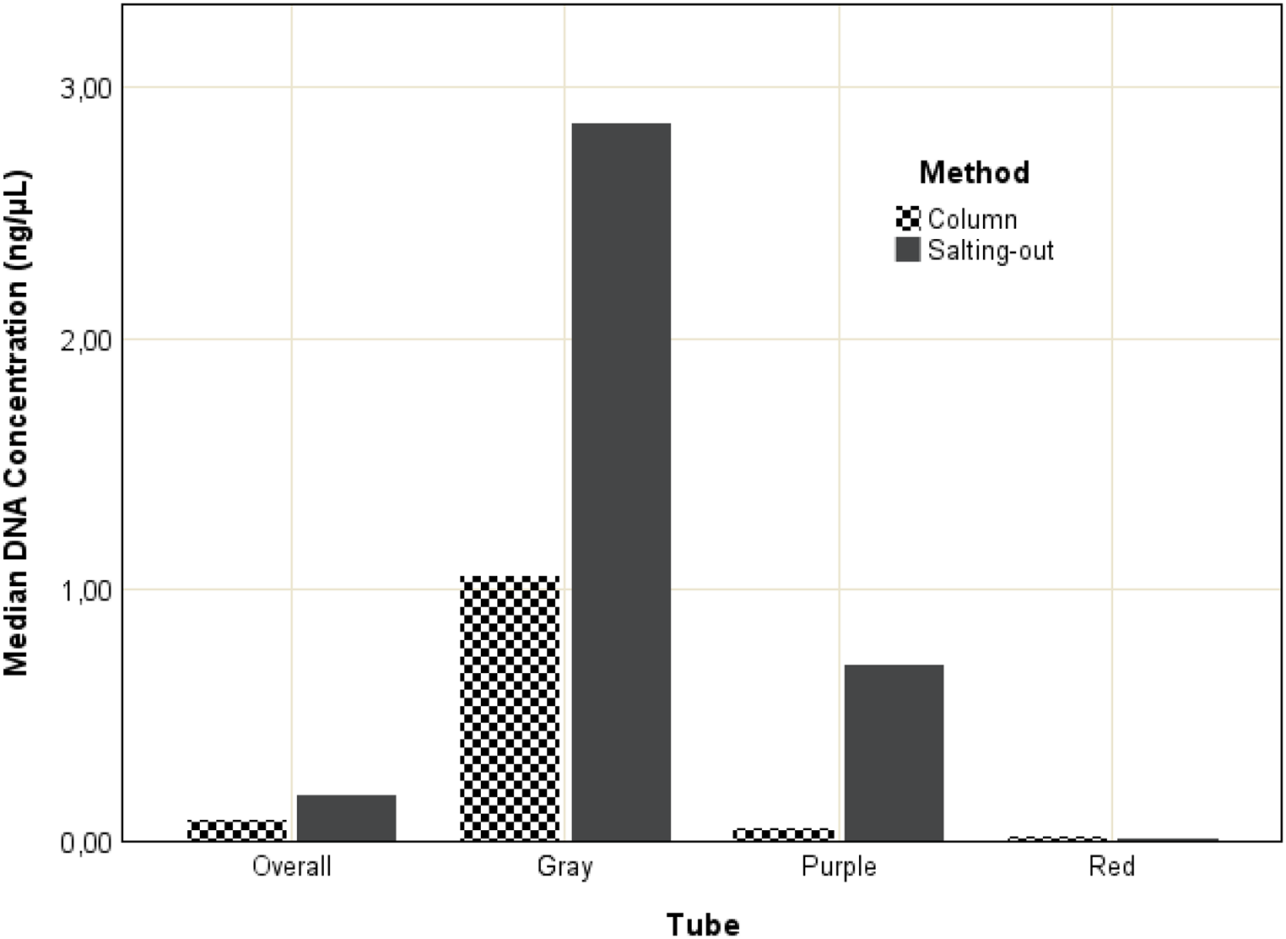
Comparison of the median DNA concentrations (ng/µL) obtained overall and within gray, purple, and red-top tubes following DNA extraction using a column-based and modified salting-out method following a five-year storage period. DNA concentrations were determined using real-time PCR

Overall, the modified salting-out method significantly increased the median DNA concentration from 0.08 ± 8.02 ng/µL to 0.18 ± 401.43 ng/µL (Z = 5.015; *r* = 0.483; *p* < 0.001) following a five-year storage period. Median DNA yields increased from 1.05 ± 11.17 ng/µL to 2.86 ± 481.17 ng/µL in gray-top tubes (Z = 3.708; *r* = 0.553) and from 0.05 ± 5.26 ng/µL to 0.7 ± 576.27 ng/µL in purple-top tubes (Z = 3.509; *r* = 0.785), and these increases were significant (*p* < 0.001 and *p* < 0.001, respectively). Median DNA yields decreased from 0.02 ± 0.18 ng/µL to 0.01 ± 1.48 ng/µL in red-top tubes (Z = 1.22; *r* = 0.186), but this decrease was insignificant (*p* = 0.223). Median concentrations are depicted in Fig. 6. Data from samples cleaned with sodium acetate were excluded from the comparison.

### PCR amplification

Samples extracted from 22G-Tox using the column-based (Fig. 9a) and salting-out (Fig. 9b) methods visually amplified better than the 22R-Tox samples at the five-year time point. All targets amplified equally well for the 22G-Tox samples, regardless of the extraction method used. For the 22R-Tox samples, all targets could be amplified using DNA extracted with the column-based method (Fig. 9c). Amplification of targets A and C improved following repeated reactions with the PCR product as template. Targets D and F could not be amplified using DNA extracted with the salting-out method (Fig. 9d).

### Sanger sequencing

**Fig. 7:**
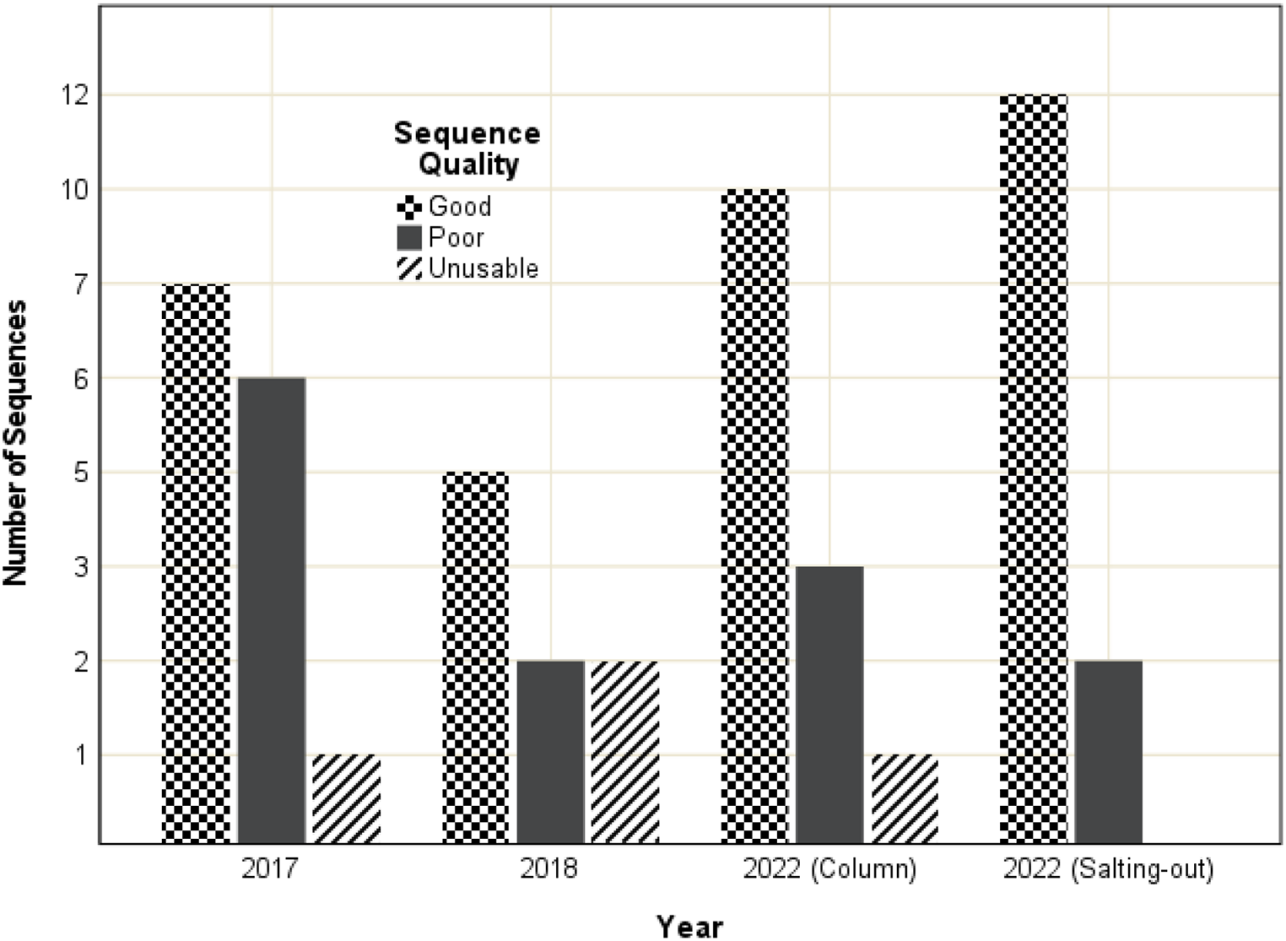
PCR amplification of the nine *CYP2D6* exons using DNA extracted from whole blood using column-based and modified salting-out methods. The whole blood was collected from Case 22 and stored in gray and red-top tubes that have undergone toxicology preparation. **a**: Amplification of DNA from the gray-top tube extracted with the column-based method. **b**: Amplification of DNA from the gray-top tube extracted with the modified salting-out method. **c**: Amplification of DNA from the red-top tube extracted with the column-based method. **d**: Amplification of DNA from the red-top tube extracted with the modified salting-out method. In each image, the wells were loaded as follows: M: Quick-Load^®^ Purple 50 bp DNA Ladder (New England Biolabs, Massachusetts, USA). A-I: *CYP2D6* gene amplification targets A-I

The 15 Sanger sequences generated in 2022 were aligned and the quality of the electropherograms were compared to those produced previously (15 sequences from 2017 and nine sequences from 2018). The sequence quality of 22G-Tox showed improvements over time (Fig. 7). In 2017, seven sequences were of good quality with one unusable sequence. In 2018, the quality of two sequences improved, while the quality of two separate sequences declined to become unusable. Ten sequences showed good quality in 2022 with one unusable sequence. Quality further improved when DNA was extracted using the modified salting-out method, where 12 sequences were of good quality with no unusable sequences.

**Fig. 8:**
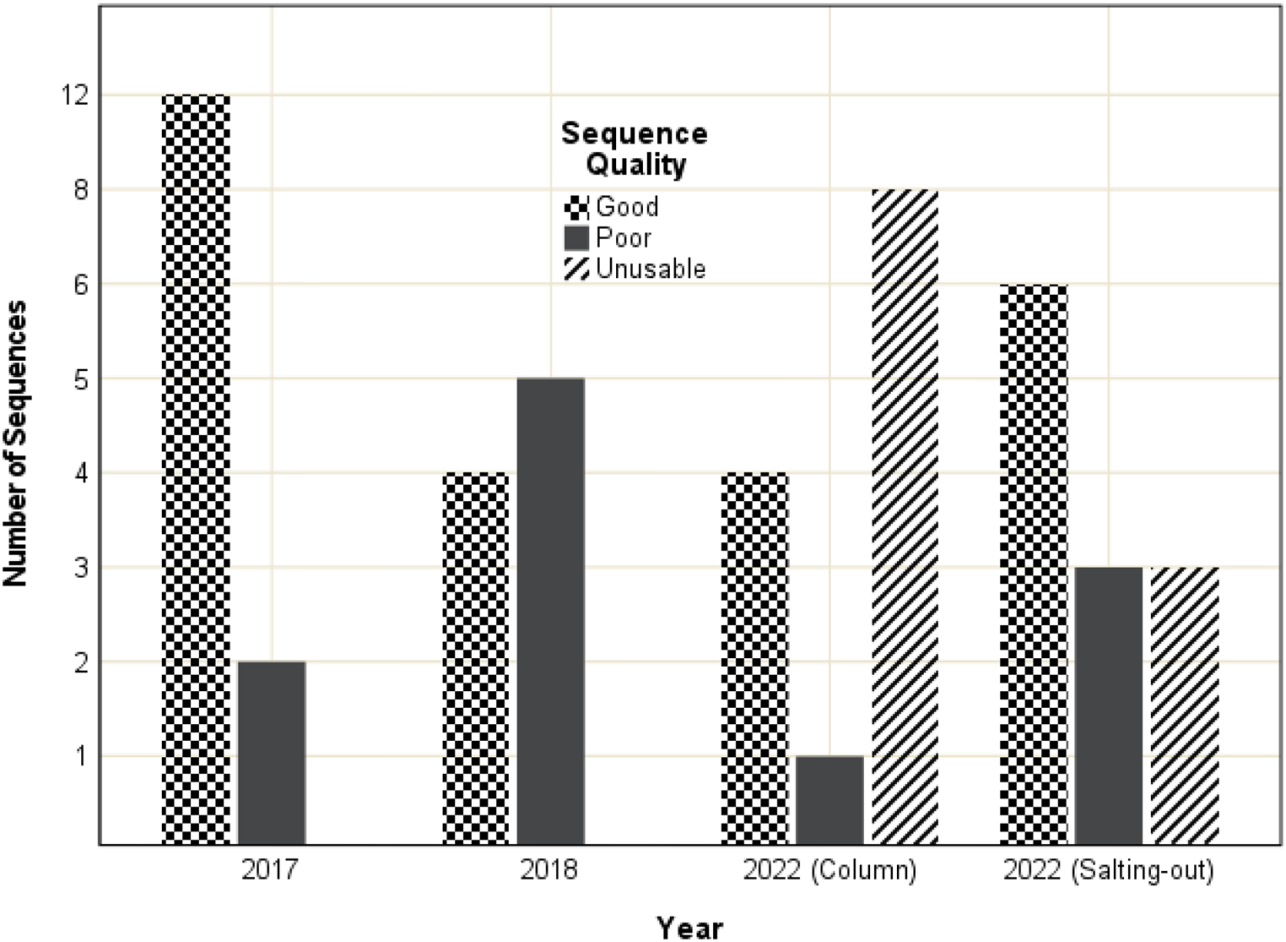
Comparison of electropherogram quality observed for 15 *CYP2D6* amplification targets between 2017, 2018 and 2022 and between DNA extracted with a column-based and modified salting-out method from the gray-top tube sample that had undergone toxicological preparation for Case 22

Sequence quality from the 22R-Tox sample declined over time (Fig. 8). In 2017, 12 sequences were of good quality with no unusable sequences. In 2018, three of the good quality sequences declined to poor quality, still with no unusable sequences. By 2022, eight sequences were unusable, and one region could not be sequenced. Quality improved (compared to the column-based method) when DNA was extracted using the modified salting-out method, where the number of good quality sequences increased from four to six and the number of unusable sequences was reduced to three (despite two targets not being sequenced due to a lack of PCR amplification).

**Fig. 9:**
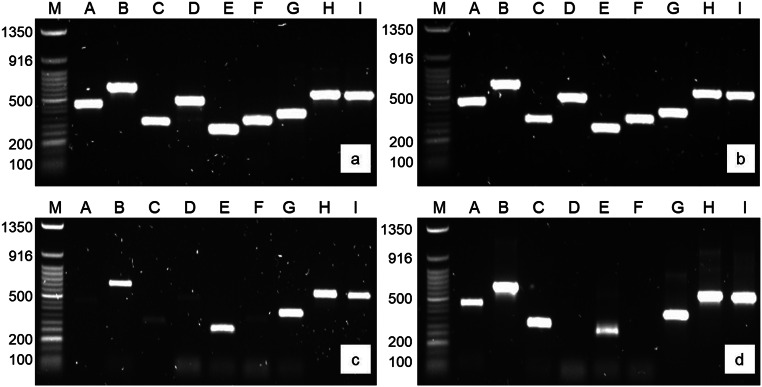
Comparison of electropherogram quality observed for 15 *CYP2D6* amplification targets between 2017, 2018 and 2022 and between DNA extracted with a column-based and modified salting-out method from the red-top tube sample that had undergone toxicological preparation for Case 22

For all experiments, positive and negative controls performed as expected.

## Discussion

In this longitudinal study, we investigated whether blood specimens that were collected in gray- and red-top tubes and had undergone toxicological sampling for drug screening would still be viable for genetic testing after a prolonged storage period. This was performed by comparing the DNA quality and quantity from these tube types at various time points, including 16 weeks, one year and five years after sample collection.

Real-time PCR was used to determine the DNA concentration and DI (Figs. 2 and 4). Gray-top tubes showed the highest median DNA concentration of 0.58 ng/µL after 5 years of storage, which was significantly higher than the red- and purple-top median concentrations (*p* < 0.001). Gray-top tubes also showed the largest number of undegraded samples and the lowest median DI of 1.19, indicating no degradation. Purple-top EDTA tubes are regarded as the gold standard to collect blood for genetic analysis [17]. However, these tubes produced suboptimal DNA yields of 0.05 ng/µL when extracted with the column-based method at the five-year time point. The median DI in EDTA tubes (1.49) also suggested slight degradation. Palmirotta et al. [26] similarly demonstrated that blood stored in fluoride oxalate tubes showed a smaller percentage decrease in DNA yield when stored at room temperature (24.84%) and 4 °C (17.73%) compared to blood stored in EDTA at the same temperatures (34.08% and 27.40%, respectively) for seven days.

The overall decrease in DNA yield, increase in DI, and superior performance of gray-top tubes may be explained by the presence of microorganisms, which could have entered the tubes following the previous opening of the samples, or following expiry of the tube caps over time. Microbes have been shown to induce DNA damage and incomplete DNA repair in mammalian cells [27]. Furthermore, sodium fluoride concentrations above 500 µM may damage DNA through genotoxicity and DNA strand breaks, as shown by Podder et al. [28] and Yüksek et al. [29]. Studies have, however, demonstrated that sodium fluoride also inhibits the growth and activity of microbes such as *Candida albicans* and *Escherichia coli*, and prevents the putrefaction of blood [30, 31]. Although sodium fluoride might affect DNA yield and integrity, it may also protect against further degradation by microbial activity, a factor lacking in the purple- and red-top tubes. However, the sodium fluoride concentration in the gray-top tubes used throughout this study was 0.1% (w/v), instead of the recommended percentage of 0.5-2% for post-mortem specimens [10]. Therefore, it is possible that this amount of sodium fluoride was not enough to have a significant negative effect on DNA, while still providing protection against microbial activity, thereby leading to the better performance of gray-top tubes. Microbe-induced DNA damage might also explain why red-top tubes had the lowest median DNA yield (0.02 ng/µL) and highest median DI (1.55), as these tubes do not contain any anticoagulants or preservatives. Consequently, there is no protection against DNA damage induced by the possible influx of microorganisms into the tube.

Another explanation for the decreased DNA yields and increased DI may be because the 4 °C temperature utilized in this study is commonly used for shorter periods, with − 20 °C or -70 °C being the optimal temperature for long-term storage [17]. Bulla et al. [32] observed decreased DNA quantities when whole blood was stored in EDTA at 4 °C for one year, which was not seen with − 20 °C or -80 °C storage temperatures. These conditions however did not affect DNA integrity [32]. Similarly, Johann et al. [33] found no correlation between DI and time when whole blood was stored at 4 °C in EDTA for the same duration of storage. The current study period was, however, longer and showed a significant time-dependent change in DNA yield and integrity.

As a quality control measure, DNA profiling was performed on all samples that underwent toxicological analysis to detect possible DNA contamination. The results indicated that no DNA contamination occurred in any of the samples, and that full profiles could be obtained from both red- and gray-top tube samples. It is crucial that samples remain free of extraneous human DNA, as the genetic analysis is centred on determining the metabolic phenotype of a decedent through the sequencing of their DNA. DNA contamination remains a likelihood in DNA-uncontrolled laboratories, since samples are handled by personnel throughout the testing process. As such, we recommend that DNA profiling be included in the genetic workflow in order for all identified variants to be attributed to the decedent in question, and not laboratory personnel.

Sanger sequencing was the culmination of the genetic assay; therefore, these results carry the most weight in addressing the aim of the study. For the gray-top tube sample, bands of high intensity were produced for all nine amplification targets, regardless of the extraction method used (Fig. 7). This is likely a result of PCR optimization in the current study. The increased PCR product yield likely had a positive effect on sequence quality, which remained good and even improved over time (Fig. 8). Sodium fluoride was, however, hypothesized to be the cause of poor sequence quality in gray-top tubes in 2017. Possible time-dependent degradation of sodium fluoride in the tubes could have minimized a reaction between this additive and the reagents, leading to increased amplification efficiency. Sequence quality was, however, better in two sequencing targets for the sample extracted with the salting-out method. This sample showed higher purity scores compared to the sample extracted with the spin column (results not shown), possibly reducing PCR inhibition, and leading to improved results.

Although higher-intensity bands were observed for targets amplified with DNA extracted from the red-top tube (likely due to PCR optimization), amplification was suboptimal for the remaining targets (Fig. 7). Certain targets that were larger in size amplified better than some smaller targets, indicating that DNA degradation did not play a role in amplification efficiency. PCR product yield had no positive impact on sequence quality and many sequences were unusable, likely due to extremely low DNA yields (Fig. 9). Andresen et al. [18] experienced similar genotyping difficulties when post-mortem whole blood was stored without additives for four years. Furthermore, DNA extracted with the salting-out method visually amplified better and had slightly improved sequence quality compared to DNA extracted with the column-based method. However, two targets failed to amplify, even after repeating the PCR reaction. Although the concentration of DNA extracted with the salting-out method was higher, it remained extremely low and proved largely insufficient for PCR-based analyses (Fig. 5).

Certain sequencing targets, however, showed poor results regardless of time, vial type or extraction method, indicating that the issue is region-specific. The *CYP2D6* gene is notoriously difficult to analyze, largely due to the presence of homologous pseudogenes [34]. Amplification and sequencing success may be improved using long-range PCR and long-read sequencing assays [35]. However, this proved problematic at the one-year time point, where longer amplification targets were thought to contribute to decreased electropherogram quality, suggesting that this might not be a solution when using old and/or degraded samples.

This longitudinal study is the first to highlight several important factors regarding the usefulness of the studied collection tubes over time. As mentioned previously, purple-top EDTA tubes are regarded as the gold standard blood collection tubes for genetic analysis [17]. Standard practice recommends that if DNA is not extracted from these tubes immediately, a buffy coat is prepared instead of storing the whole blood. As such, very little data exists about the quality and quantity of DNA extracted from whole blood stored in EDTA tubes for extended periods, and this study is the first to provide this information at a time point of five years of storage. This study also provides new data regarding the functionality of alternative vacutainers for genetic analysis, as well as the usability of samples that have undergone toxicological analysis, which may be applied to both clinical and forensic settings. If samples are only stored for a period of weeks after sample collection, it is preferable to use DNA extracted from blood stored in red-top tubes due to the superior quality of sequences obtained. However, if circumstances like processing backlogs necessitate that samples be stored for a matter of years, gray-top tubes should be the sample of choice. These results indicate the viability of post-mortem genetic analyses and provide critical information to aid in the generation of a standardized protocol that can be implemented in forensic mortuaries.

Certain limitations must be noted. The stipulated exclusion criteria are often encountered in a forensic setting, but the results of this study cannot be extrapolated to these cases. Therefore, it remains unknown whether blood samples collected from these cases can yield suitable DNA under the same parameters. Although 30 cases conferred statistical significance, the small sample size may not be fully representative of the larger population. Finally, directly contrasting the final eluted DNA concentrations recovered using the two extraction methods does not provide an equal comparison, due to the differing sample input volumes used and the inability to increase this variable in commercial extraction kits.

Future studies may investigate other drug-carrier, and drug-metabolizing enzymes, as the metabolism of many drugs is governed by more than one enzyme. Identifying specific drugs commonly used in the population could direct investigations toward relevant enzymes. The mechanism by which various additives in blood storage tubes affect DNA, and alternative extraction methods may also be investigated.

In conclusion, the concentration and sequence quality of DNA extracted from whole blood collected in sodium fluoride/potassium oxalate or no additive and stored long-term following preparation for toxicology testing was investigated. The results of the current study were sufficient to demonstrate that DNA extracted from blood stored at 4 °C in gray-top tubes using a modified salting-out method is optimal to conduct genetic analysis after five years, and there is no need to collect an additional sample specifically for DNA testing at the time of autopsy. This is particularly beneficial in resource-constrained settings where optimal sample collection containers are not always available, and delays in sample processing are often experienced. This information is a critical step in generating a standardized protocol, which may be implemented to include genetics as a standard post-mortem practice.

## Data Availability

The datasets generated and analyzed during the current study are available in the University of Cape Town institutional repository (ZivaHub) under the following 10.25375/uct.26411089.
